# Clinical case giant malignant phyllodes tumor of the breast

**DOI:** 10.1093/jscr/rjag735

**Published:** 2026-08-23

**Authors:** Alan Hari Silva Amaya, Julio Caleb Arrieta Navarro, Álvaro M Ñaña-Córdova, Elkin J Peláez-Cruz, Sorely Pacovilca Chura

**Affiliations:** Department of Surgery and Specialties, Oncological Surgery Service, Hospital II-2 Tarapoto, San Martín, Peru; Faculty of Human Medicine, National University of San Martín, Tarapoto, Peru; Faculty of Human Medicine, National University of San Martín, Tarapoto, Peru; Faculty of Human Medicine, Scientific University of the South, Lima, Peru; Faculty of Medicine, National University of Trujillo, La Libertad, Peru; Scientific Society of Medical Students of the National University of Trujillo, La Libertad, Peru; Faculty of Human Medicine, Peruvian Union University, Lima, Peru

**Keywords:** malignant phyllodes tumor, total mastectomy, rare breast neoplasm, fibroepithelial tumors, oncologic surgery

## Abstract

Malignant phyllodes tumor is a rare and aggressive breast neoplasm with the potential for rapid and recurrent growth. We present the case of a 56-year-old woman from San Martín, Peru, with a left breast mass exhibiting exponential growth, ulceration, necrosis, and asymmetry. Physical examination revealed a multinodular, purplish-red, indurated, and slightly painful mass. Ultrasound showed a 12-cm hypoechoic, heterogeneous mass with areas of necrosis, increased vascularity, and contact with the muscle layer (Breast Imaging Reporting and Data System category 4). Core biopsy revealed a fibroepithelial neoplasm with high mitotic activity (10/10 HPF). The patient underwent left total mastectomy with subsequent margin extension and partial-thickness skin grafting, achieving complete resection and satisfactory recovery. This case highlights the challenges in the diagnosis and surgical management of this pathology in resource-limited settings where timely surgery plays a crucial role in treatment and prevents recurrence.

## Introduction

Phyllodes tumor (PT) is a rare fibroepithelial breast neoplasm, accounting for <1% of all breast tumors [1, 2], with an estimated incidence of 2.1–2.6 cases per million women annually [3]. It occurs most frequently between 40 and 50 years of age and is classified as benign, borderline, or malignant according to histopathological features, including stromal cellularity, mitotic activity, nuclear atypia, tumor margins, and stromal overgrowth [4].

Malignant PTs constitute only 10%–15% of all PTs and represent an exceptionally uncommon subgroup of breast neoplasms, with an estimated incidence of 0.5–0.9 cases per million women per year [5–7]. Giant malignant PTs, generally defined as lesions larger than 10 cm, are particularly rare and pose significant diagnostic and therapeutic challenges due to their rapid growth, local aggressiveness, risk of recurrence, and potential for distant metastasis [4, 8].

These tumors typically present as a painless, rapidly enlarging breast mass with possible deformity, ulceration, or skin necrosis. Axillary involvement is uncommon due to predominantly hematogenous spread [3, 7].

Diagnosis is challenging because of its similarity to other rapidly growing breast neoplasms. Imaging studies, including ultrasound and magnetic resonance imaging, can aid in characterization; however, definitive diagnosis relies on histopathological examination [8, 9].

Complete surgical excision with negative margins is the standard treatment. Giant tumors frequently require mastectomy and reconstruction, while radiotherapy may be considered in selected cases to reduce local recurrence [6, 10].

## Case report

A 56-year-old woman from Saposoa, San Martín, Peru, was referred to the surgical oncology service for evaluation of a rapidly enlarging left breast mass with a 1-year history of progressive growth. Physical examination revealed a multinodular mass measuring 25 × 20 cm, involving all breast quadrants and displaying a reddish-purple appearance. The nipple–areolar complex was preserved, and a 1.2-cm ipsilateral axillary lymph node was palpable. The patient denied a family history of breast cancer or PT. She also had mild intellectual disability, resulting in communication and functional limitations.

**Figure 1:**
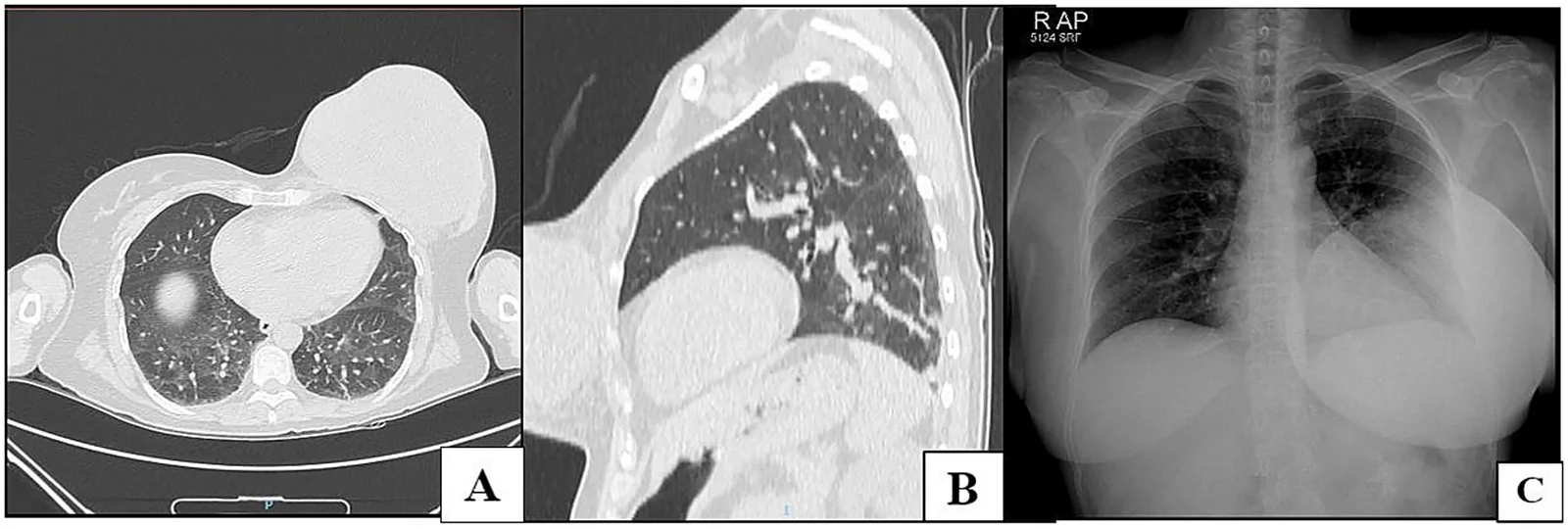
The radiological and macroscopic findings of the tumor; computed tomography revealed a large left breast mass with lobulated and irregular margins, areas of central necrosis, neovascularization, and probable involvement of the adjacent muscle layer; likewise, the clinical and surgical specimen images demonstrate the enormous size of the lesion and the significant local involvement caused by tumor growth.

Breast ultrasound demonstrated a heterogeneous hypoechoic mass with lobulated margins, measuring 120 × 69 mm, containing focal necrotic areas and increased vascularity, along with reactive axillary lymph nodes. Chest computed tomography revealed a 126 × 142 × 103 mm left breast tumor with lobulated irregular borders, central necrosis, peripheral contrast enhancement, and prominent neovascularization. The lesion infiltrated the nipple–areolar complex and was closely related to the pectoralis major and adjacent intercostal muscles (Fig. 1).

Core needle biopsy showed a fibroepithelial neoplasm with increased stromal cellularity, marked atypia, necrosis, and a mitotic count >10/10 high-power fields, raising suspicion of malignant PT (Table 1). A left total mastectomy was performed. Histopathological examination confirmed a 15-cm malignant PT characterized by marked stromal cellularity and atypia, stromal overgrowth, skin infiltration by direct extension, mitotic activity >10/10 high-power fields, absence of heterologous elements, and negative surgical margins (Fig. 3A–C).

**Table 1:** Characteristics of PT types.

Feature	Benign	Borderline	Malignant
Stromal cellularity	Low	Moderate	High
Nuclear atypia	Absent or mild	Moderate	Marked
Mitotic index	<5 mitoses per 10 high-power fields	5–9 mitoses per 10 fields	≥10 mitoses per 10 fields
Tumor margins	Well-defined	They can be infiltrative	Generally infiltrative
Metastatic potential	Absent	Low	High, mainly hematogenous

Summarizes the main histopathological characteristics used to classify PTs as benign, borderline, or malignant. Among the most relevant criteria are stromal cellularity, the degree of nuclear atypia, mitotic activity, and the pattern of tumor margins. In our case, the presence of marked stromal hypercellularity, significant atypia, and mitotic activity greater than 10 mitoses per 10 high-power fields allowed us to classify the lesion as a malignant PT. Source: Adapted from Tan *et al*. [1] and Li *et al*. [8].

**Figure 2:**
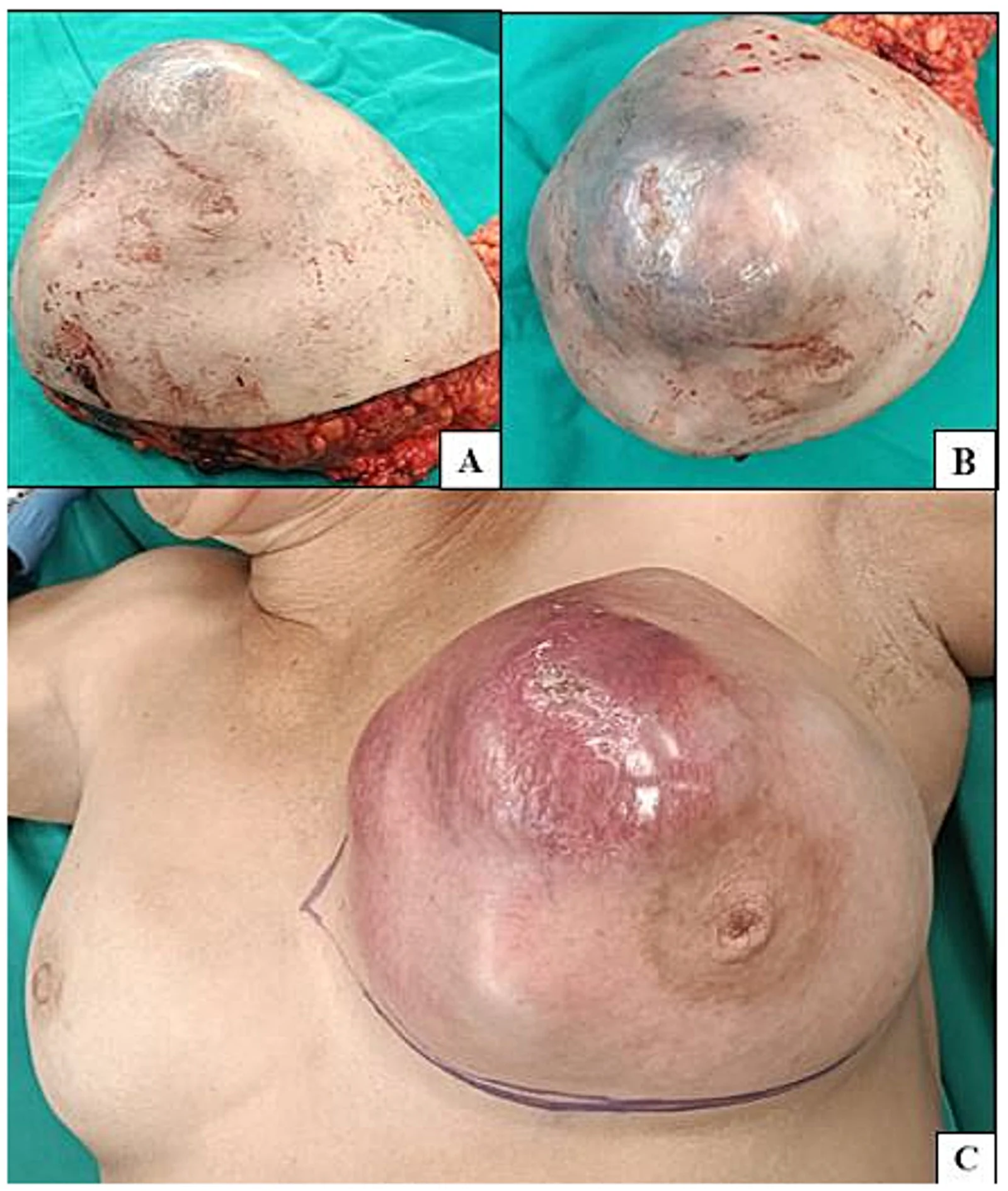
The characteristic microscopic findings of the PT, highlighting the progressive increase in stromal cellularity, the presence of nuclear atypia, and the typical cleft or “leaf” configuration, findings that led to the diagnosis of a fibroepithelial neoplasm with aggressive biological behavior.

The surgical specimen weighed 1.526 kg and measured 25 × 20 × 12 cm, containing a solid mass measuring 15 × 11.5 × 9 cm involving all breast quadrants and the retroareolar region (Fig. 2). Owing to the extensive defect after resection, primary closure was not feasible, leaving a 12 × 6 cm central defect. Following intraoperative margin widening, a split-thickness skin graft was placed 10 days later after adequate granulation tissue formation. At 7-day follow-up, the graft remained viable with satisfactory integration and coloration (Fig. 4).

**Figure 3:**
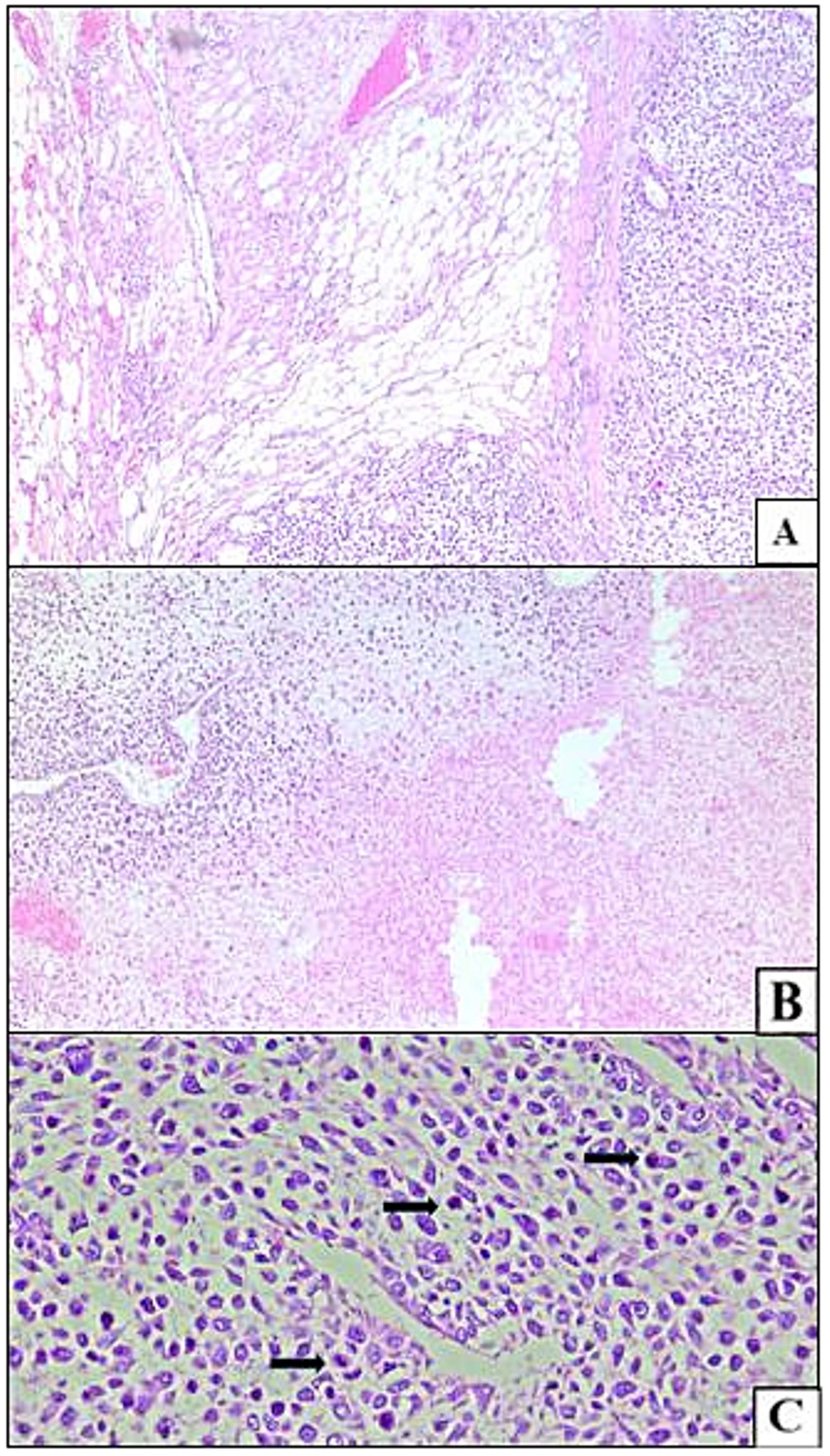
The histopathological criteria for malignancy observed in the surgical specimen, including infiltrative margins, areas of tumor necrosis, marked stromal hypercellularity, nuclear pleomorphism, and high mitotic activity; these findings confirmed the definitive diagnosis of malignant PT.

**Figure 4:**
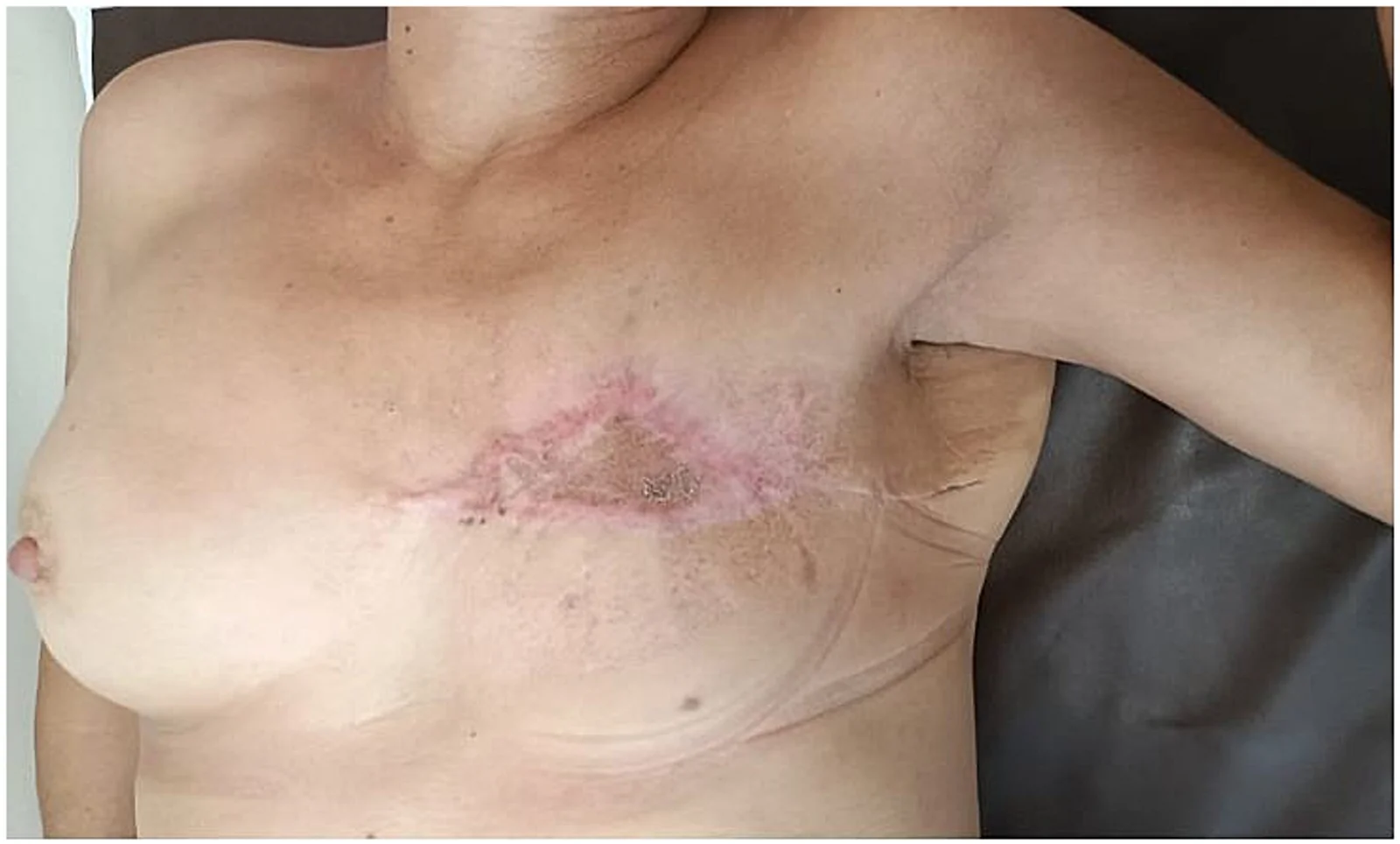
The postoperative evolution after total mastectomy and reconstruction using a partial-thickness skin graft; adequate graft integration, satisfactory coverage of the surgical defect, and absence of evident local complications during follow-up are observed.

## Discussion

PTs account for <1% of all breast neoplasms, and only 10%–15% are classified as malignant, making giant malignant PTs exceptionally uncommon. Their rarity limits the availability of robust evidence and poses challenges in diagnosis and management, particularly in resource-limited settings [9, 10].

Our patient presented with a giant malignant PT measuring 15 cm on histopathological examination, associated with skin infiltration and rapid growth. These features are recognized markers of locally aggressive disease and have been associated with an increased risk of local recurrence and distant metastasis [3]. As reported in previous studies, differentiating malignant PTs from other rapidly growing breast lesions remains difficult because clinical and imaging findings are often nonspecific.

In this case, ultrasonography identified a suspicious fibroepithelial lesion but underestimated its biological aggressiveness. Conversely, computed tomography demonstrated central necrosis, neovascularization, and close contact with the pectoral and intercostal muscles, findings that influenced surgical planning. Although preoperative core needle biopsy suggested a malignant fibroepithelial neoplasm, definitive diagnosis and risk stratification were only achieved after histopathological evaluation of the surgical specimen. This highlights the limitations of preoperative assessment and the importance of integrating imaging, pathology, and clinical findings when planning treatment [4].

Complete surgical excision with negative margins remains the cornerstone of treatment for malignant PTs. In our patient, total mastectomy achieved clear margins, although subsequent widening of the deep margin was performed because of its proximity to the underlying muscle. Given the uncertainty regarding margin status and the moderate size of the postoperative defect, delayed reconstruction with a split-thickness skin graft was selected. This strategy provided satisfactory wound coverage while allowing confirmation of adequate oncological control before definitive closure.

A limitation of this report is its single-case design and short follow-up. However, 6-month postoperative computed tomography showed no local recurrence or distant disease, and no adjuvant therapy was needed. This case demonstrates that giant malignant PTs can be successfully managed in resource-limited settings, with delayed skin graft reconstruction serving as an effective alternative when advanced reconstructive options are unavailable.

## Notes

This work was presented and discussed at the International Congress on Cancer and Public Health 2024, organized by Ricardo Palma University, Regional Council III Lima, and Fundashon Prevenshon Curacao in Lima, Peru, 2024.
